# Pharmacologic Sedation–Related Respiratory Complications in Pediatric Dentistry: A Decade-Long Scoping Review

**DOI:** 10.5812/ijpr-170313

**Published:** 2026-06-06

**Authors:** Leila Mohammadpour-Belvirdy, Elham Sadati, Maryam Hassanzad, Ali Valinejadi

**Affiliations:** 1 Pediatric Respiratory Diseases Research Center, National Research Institute of Tuberculosis and Lung Diseases, Shahid Beheshti University of Medical Sciences, Tehran, Iran; 2 Department of Pediatrics, Fakeeh University Hospital, Dubai, United Arab Emirates; 3 Khomein University of Medical Sciences, Khomein, Iran

**Keywords:** Procedural Sedation, Pediatric Dentistry, Respiratory Complications, Hypnotics And Sedatives, Drug-related Side Effects And Adverse Reactions

## Abstract

**Context:**

Procedural sedation is widely used in pediatric dentistry to facilitate dental care in uncooperative children. From a pharmacological perspective, sedative agents differ substantially in their mechanisms of action, routes of administration, and safety profiles. Among the reported adverse effects, respiratory complications remain the most clinically significant and potentially life-threatening. Despite extensive clinical use, a comprehensive synthesis of respiratory adverse events (RAEs) associated with commonly used sedative drugs in pediatric dental settings is lacking. This scoping review aimed to map the existing evidence on respiratory complications associated with pharmacological sedation in pediatric dentistry.

**Evidence Acquisition:**

A scoping review was conducted in accordance with established methodological frameworks. Electronic databases were systematically searched for studies reporting the use of sedative or anesthetic agents in pediatric dental procedures. Eligible studies included clinical trials, cohort studies, case series, and retrospective analyses that documented respiratory outcomes. Data were extracted on patient characteristics, sedative agents and combinations, dosages, routes of administration, monitoring methods, and reported respiratory complications.

**Results:**

The included studies showed substantial variability in sedation protocols. The most frequently reported agents were benzodiazepines, chloral hydrate, ketamine, propofol, dexmedetomidine, and opioid combinations. For clarity, respiratory complications in this review are categorized as mild (e.g., transient oxygen desaturation, partial airway obstruction), moderate (e.g., hypoventilation requiring intervention), and severe (e.g., laryngospasm, apnea requiring advanced airway management). RAEs ranged from mild, transient events (e.g., oxygen desaturation and airway obstruction) to more serious conditions, including laryngospasm and apnea. Most complications were managed successfully with basic airway maneuvers, supplemental oxygen, or brief positive-pressure ventilation. Severe outcomes requiring advanced airway intervention were rare. Continuous respiratory monitoring, particularly pulse oximetry and capnography to facilitate earlier detection of ventilatory compromise, was consistently associated with prompt management of adverse events.

**Conclusions:**

Pharmacological sedation in pediatric dentistry is generally safe when appropriate drug selection, dosing, and monitoring are used. Respiratory complications are relatively common but are typically mild and reversible. From a pharmacological perspective, careful consideration of sedative combinations, cumulative respiratory depressant effects, and vigilant monitoring is essential to optimize patient safety. Further high-quality studies are needed to refine evidence-based sedation protocols and minimize respiratory risks.

## 1. Context

Pharmacological management of anxiety and cooperation is essential for the safe performance of complex pediatric dental procedures (1, 2). Although these agents facilitate treatment at the interface of dentistry and pharmacotherapy, respiratory adverse events (RAEs) remain the most critical adverse reactions (3-5). These complications span a spectrum from mild events, such as oxygen desaturation and airway obstruction, to severe conditions, including apnea and laryngospasm. RAEs, including hypoxia, airway obstruction, and laryngospasm, are direct consequences of sedative pharmacodynamics. Children are uniquely vulnerable to drug-induced respiratory compromise because of anatomical and physiological characteristics, such as smaller airway diameters and higher metabolic oxygen demands (6, 7). Systematic reviews confirm that airway complications are the primary cause of morbidity during pediatric sedation, necessitating rigorous pharmacovigilance (8-10).

Different drugs exhibit distinct safety profiles. Midazolam, the most commonly used pediatric benzodiazepine, induces dose-dependent respiratory depression through potentiation of GABA activity, potentially leading to hypoventilation or transient obstruction (6, 11-13). Ketamine offers bronchodilatory advantages for asthmatic children; however, desaturation or apnea can occur, particularly during prolonged procedures or when combined with other agents (8, 14). Remimazolam, a recently introduced short-acting benzodiazepine with favorable pharmacokinetics, still carries a risk of hypoventilation, necessitating continuous monitoring (2, 15, 16).

Reported respiratory event rates vary: Huang et al. found oxygen desaturation in over 20% of patients (17), while other reports documented transient obstruction in most cases. Although such events are usually reversible with minimal intervention, serious outcomes, such as aspiration or hypoxia-related cardiac arrest, can occur, requiring constant vigilance. Furthermore, preexisting respiratory disorders, particularly asthma, increase susceptibility to sedation-related RAEs, such as bronchospasm and hypoxemia, necessitating adjustments to sedation protocols and dosing (18-20). Higher ASA status, longer treatment duration, and invasive procedures correlate with increased respiratory risk. These factors underscore the need for rational, individualized pharmacotherapy to optimize safety and efficacy.

Safe sedation requires standardized protocols, precise dosing, and rigorous monitoring (21). Guideline-adherent institutions have been shown to have lower complication rates, while other reports emphasize pulse oximetry and capnography for early detection (22). These pharmacovigilance principles require that adverse reactions be anticipated, promptly detected, and systematically reported (3, 15).

The clinical setting is critical: while hospitals can manage airway emergencies effectively, outpatient dental clinics often lack comparable resources, heightening the need for prevention and early detection in non-hospital environments (23).

Syndromic children and those with comorbidities face disproportionately higher risks, requiring tailored pharmacological planning (19). However, inconsistent RAE definitions and reporting standards hinder comparisons across studies and limit generalizability (3, 24). Furthermore, the scarcity of prospective multicenter investigations, with most evidence being retrospective, limits the development of universal safety guidelines and the definition of clear pharmacological risk thresholds (25-28).

Balancing sedation efficacy with safety remains challenging. Nitrous oxide may provoke airway irritation in sensitive children, while potent agents such as propofol and remimazolam achieve rapid sedation but carry a risk of dose-dependent respiratory depression, necessitating strict monitoring (1, 15). The literature consistently recommends rational drug use, individualized dosing, thorough preoperative screening, and advanced monitoring to mitigate risks (3, 15, 27, 29).

In summary, RAEs are the primary adverse drug reactions in pediatric dental sedation, arising from interactions among drug effects, patient vulnerability, and procedural conditions. This scoping review synthesizes evidence on drug-related respiratory events to identify recurring pharmacological themes and knowledge gaps. Ultimately, it aims to assist clinicians and pharmaceutical scientists in promoting safer and more rational drug use for children undergoing dental treatment.

## 2. Evidence Acquisition

### 2.1. Study Design

This scoping review follows the Arksey and O'Malley framework, refined by Levac et al. (30), and adheres to the PRISMA-ScR guidelines. This methodology was selected to systematically map the extent, range, and nature of the evidence on RAEs associated with sedative drugs in pediatric dentistry.

### 2.2. Research Questions

This review addresses the types of respiratory complications in pediatric sedation, the associated pharmacological agents, risk factors, and mitigation strategies. These questions follow the PCC framework: Population (P), children and adolescents (≤18 years) undergoing dental treatment; Concept (C), RAEs (e.g., hypoxia, apnea, laryngospasm, bronchospasm, airway obstruction) linked to sedative drugs; and Context (C), outpatient clinics, hospitals, or academic settings.

### 2.3. Eligibility Criteria

The inclusion criteria comprised studies involving patients ≤ 18 years undergoing dental sedation that reported RAEs (hypoxia, hypoventilation, apnea, laryngospasm, bronchospasm, airway obstruction, or aspiration). Eligible study designs included clinical trials, observational studies, systematic or narrative reviews, and case reports or case series. The exclusion criteria comprised studies focused on adults, non-sedative use, or outcomes unrelated to RAEs. Articles addressing dental procedures without reference to pharmacological sedation were also excluded.

### 2.4. Information Sources and Search Strategy

A comprehensive search was conducted in PubMed/MEDLINE, Scopus, Web of Science, the Cochrane Library, and Google Scholar (for grey literature). Studies published from 2016 to 2025 were included. The search used MeSH terms and keywords, including "pediatric dentistry," "sedation," and "RAEs" (e.g., hypoxia, apnea, airway obstruction), combined with Boolean operators.

### 2.5. Selection of Sources of Evidence

All references were managed in EndNote/Zotero, and duplicates were removed. Two independent reviewers screened titles and abstracts, followed by full-text evaluation based on the eligibility criteria. Disagreements were resolved through discussion or consultation with a third reviewer to reach consensus.

### 2.6. Data Charting Process

Data were extracted using a standardized form. Key information included authorship, publication year, country, study design, and sample characteristics. Specific data on sedative types, dental procedures, and RAEs (type, frequency, severity) were recorded, along with identified risk factors and recommended monitoring or prevention strategies.

### 2.7. Data Synthesis

Extracted data underwent descriptive and thematic synthesis rather than statistical analysis. Findings were categorized by RAE type and incidence, associated pharmacological agents, risk factors, and prevention strategies. Owing to heterogeneity in study designs, populations, and outcome definitions, a meta-analysis was not feasible; therefore, the synthesis remained qualitative.

### 2.8. Ethical Considerations

The protocol was approved by the Ethics Committee of the National Research Institute of Tuberculosis and Lung Diseases (NRITLD), Shahid Beheshti University of Medical Sciences (IR.SBMU.NRITLD.REC.1404.053). As this was a review of published literature, no primary data collection involving human participants was required.

## 3. Results

### 3.1. Selection of Sources of Evidence

**Figure 1.**
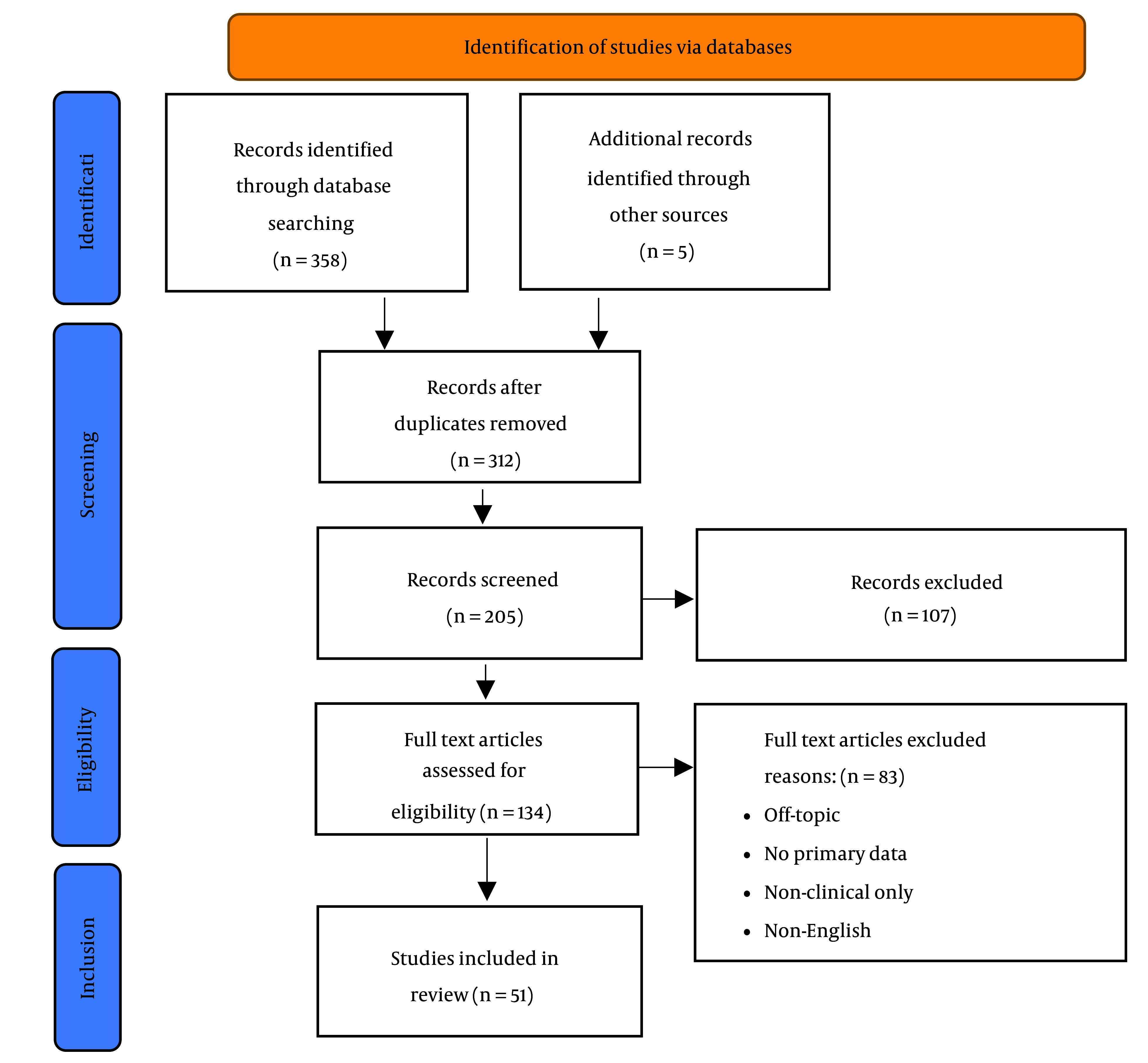
Study selection process (PRISMA-ScR flowchart)

Based on the PRISMA-ScR flowchart (Figure 1), 358 records were initially identified. After removal of 46 duplicates, 312 unique records underwent title and abstract screening, resulting in 107 exclusions. Of the 134 full-text articles assessed, 81 were excluded (reasons: off-topic, no primary data, non-clinical, or non-English). Ultimately, 51 studies were included in the review.

### 3.2. Geographic and Temporal Mapping

Analysis of 51 studies (Table 1) reveals a global research shift from Western contexts toward Asia and the Middle East between 2016 and 2025. The highest research volume originated from India, Iran, and Turkey, reflecting a focus on pharmacological sedation as a safe, cost-effective alternative to general anesthesia. Contributions from Germany, Italy, South Korea, Vietnam, and Iraq further highlight a diverse landscape, with recent clinical innovation increasingly centered in emerging medical hubs.

**Table 1.** Characteristics of Included Studies and Populations

Author (y)	Country	Study Design	Sample Size	Age range	Sex (M/F)	Patient Conditions	Setting	Type of Dental Procedure	Notes
**Vašáková et al. (2020) (42)**	Czech Republic	Retrospective Observational	n = 272 (418 sessions)	5.5y (± 1.9)	139 M / 133 F	NR	University Hospital Dept.	Fillings, Extractions, SSC, Surgery	EAPD guidelines; 9 incomplete cases; 5 Flumazenil used
**Albaker et al. (2022) (39)**	USA	Retrospective Review	n = 324 (404 sessions)	6.85y (± 2.31)	NR	Mostly ASA I-II	Hospital Dental Clinic	Exodontia (39%), Restorative	1 Adverse event (Tachycardia, resolved)
**Hammadyeh et al. (2019) (44)**	Syria	RCT	n = 40	2 - 6y (Mean: 4.0)	17 M / 23 F	ASA I	University Pediatric Clinic	Pulpotomy	No adverse/resp events; SpO_2_ > 97%
**Alzein et al. (2022) (45)**	Syria	Randomized Clinical Trial	n = 46	3 - 6y	NR	ASA I–II; no systemic diseases	NR	Dental treatments (Deep sedation)	Sevoflurane vs Propofol groups
**Thakur et al. (2021) (46)**	India	Randomized Clinical Trial	n = 36	5.5y (± 1.3)	55.6% M / 44.4% F	ASA I	Hospital Minor OT	Pulpotomy + Restorations	Vitals q15 - 20 min; SpO_2_ ≥ 93%; No resp events
**Hammadyeh et al. (2019) (47)**	Syria	Randomized Controlled Trial	n = 40	2 - 6y	19 M / 21 F	ASA I	Damascus University Clinic	Pulpotomy	No AE;SpO_2_ > 97%; Shorter recovery in Dex group (D: 10/10; K: 9/11)
**Kip et al. (2019) (40)**	Turkey	Retrospective Clinical Study	n = 553	5.99y (± 2.53)	248 M / 305 F	ASA I–III	Faculty of Dentistry Clinic	Various treatments (15 - 120 min)	Large sample size; SPSS analysis (χ^2, t-test)
**Ansari et al. (2018) (48)**	Iran	Randomized Crossover Trial	n = 26	2 - 6y	NR	ASA I (Frankl I)	Dental School Clinic	Restorative + Pulpotomy	Houpt scale used; IV sedation
**ElKhatib et al. (2024) (49)**	Egypt	Triple-blind RCT	n = 72	4 - 6y	44 M / 28 F	ASA I-II	Pediatric Outpatient Clinic	Restorations, Pulpotomy, SSC, Extractions	Nebulized Dex/Midazolam; Evaluated sedation & analgesia
**Rienhoff et al. (2022) (41)**	Germany	Retrospective Longitudinal	n = 311	74.2mo (± 24.7)	169 M / 142 F	ASA I–II	Private Pediatric Clinic	Restorations, Pulpotomy, SSC, Extractions	Midazolam + Hypnosis; up to 3 sessions
**Wu et al. (2023) (37)**	China	Retrospective	n = 342	4.9y (Median)	234 M / 108 F	ASA < III	Outpatient Pediatric Dentistry	Mixed (1 - 13 teeth)	10.2% coughing; 2155 teeth treated; incl. neuro disorders
**Binh et al. (2025) (50)**	Vietnam	Prospective Double-blind RCT	n = 80	4.7y (± 0.9)	NR	ASA I–II	Outpatient Pediatric Clinic	Extraction, RCT, Fillings, SSC	95 - 100% Success; Procedures < 30 min
**Sado-Filho et al. (2021) (33)**	Brazil	Triple-blind RCT	n = 88	18 - 87mo	50 M / 38 F	ASA I–II	University Sedation Clinic	ART (Manual caries removal)	90.9% Completed; 9% Aborted behavior-wise
**Mehran et al. (2017) (51)**	Iran	Randomized Crossover Trial	n = 17	4.5y (± 0.9)	9 M / 8 F	ASA I (Frankl II)	Dental School (Pediatric Dept)	Pulpotomy + SSC (after LA)	Two sessions; ≥1-week interval; Intranasal sedation
**Patel et al. (2025) (52)**	India	Triple-blind Parallel RCT	n = 50	4 - 8y	NR	ASA I (Frankl 1 - 2)	Pediatric Dentistry Dept	Multiple Primary Extractions	Intranasal MID vs DEX; Atomized; Fasting 6h
**Ghajari et al. (2016) (53)**	Iran	Randomized Crossover Trial	n = 16	Mean 48mo (3 - 6y)	6 M / 10 F	ASA I (Frankl 1)	University Sedation Unit	Pulpotomy + SSC + LA	2 sessions; No deep sleep; NPO 6h/4h
**Abdulhamid et al. (2016) (35)**	USA	Open-label Clinical Trial	n = 24	<10y (Mean: 4.8)	13 M / 11 F	ASA I–II (Mild asthma)	Hospital Dental Clinic	Restorations, Extractions, Pulpectomy	Chloral hydrate safety in asthmatic children
**Joshi et al. (2020) (54)**	India	Randomized Comparative Trial	n = 30	4 - 8y	NR	ASA I–II	Dental Outpatient Clinic	Restorations, Extractions, Pulp therapy	IV Ketamine-Propofol vs Ketamine-Dex; ECG/BP monitoring
**Preethy & Somasundaram (2022) (55)**	India	Split-mouth Crossover RCT	n = 35	5.66y (± 0.77)	18 M / 17 F	ASA I (Frankl 1 - 2)	Academic Dental Hospital	Bilateral Pulpectomy (IANB)	Crossover design; Physiological monitoring
**Janiani et al. (2024) (56)**	India	Randomized Crossover Trial	n = 14	6.14y (± 1.56)	7 M / 7 F	ASA NR (Healthy)	Academic Dental Hospital	Single-visit Pulpectomy	3-arm (IN DEX, IN MID, N_2O); 1-week washout
**Hamod et al. (2022) (36)**	Syria	Randomized Double-blind RCT	n = 20	7.9y (± 0.9)	NR	ASA I–II (Down Syndrome)	Damascus Univ. Clinic	Endo, Conservative, Extractions	IN DEX vs MID; Vitals every 5 min (AAPD)
**Arnaout et al. (2025) (57)**	Syria	Single-blind Comparative RCT	n = 40	4.8y (± 0.9)	NR	ASA I–II	University Dental Clinic	Pulpotomy + SSC	Comparison study (Lower molar treatment)
**Zouaidi et al. (2022) (24)**	USA	Retrospective Observational	n = 690	7.4y (± 4.1)	358 M / 332 F	ASA I–II (100%)	Academic Dental School	Routine Care (Sedation)	EHR-based AE monitoring; Avg 2 visits/child
**Yinger et al. (2024) (34)**	USA	Retrospective Chart Review	n = 82	10.7y (± 2.2)	32 M / 50 F	ASA I-II (9% Dev. Disability)	Academic Sedation Clinic	Permanent 1st Molar Extraction	Anxiety/Cooperation focus; Reasons for sedation
**Razavi & Malekianzadeh (2022) (58)**	Iran	Retrospective Cohort	n = 250	3.7y (2.5 - 5)	170 M / 80 F	ASA I–II	Pediatric Hospital Dental Office	Multiple procedures (Deep sedation)	---
**Unkel et al. (2021) (59)**	USA	Retrospective Chart Review	n = 149	4.6y (± 1.0)	63 M / 86 F	ASA I–II (Extensive exclusion)	Hospital Pediatric Sedation	Restorations, Pulp, Extractions	DEX+N_2O vs MID+N_2O vs MID+HYD+N_2O
**Liu et al. (2025) (38)**	China	Retrospective Cohort	n = 513	4.80y (± 1.27)	359 M / 154 F	ASA I–II (6.8% Autism)	Academic Hospital	Treatments < 2h	ROC cutoff 79 min for AE prediction
**Gomes et al. (2017) (60)**	Brazil	Triple-blind RCT Protocol	n = 84	2 - 6y	NR	ASA I–II (Low airway risk)	Academic Dental Sedation Center	Restorative (under LA)	NCT02447289; 3-arm comparison
**Kocaoğlu et al. (2025) (43)**	Turkey	Retrospective Cross-sectional	n = 504	5.8y (± 1.7)	259 M / 245 F	ASA I–III	Academic Hospital	Restorative, Endo, Extractions	STROBE guidelines; Hypoxemia risk analysis
**Wang et al. (2023) (61)**	China	Prospective Cohort	n = 60	51.5mo (Median)	27 M / 33 F	ASA I–II	Outpatient Dental Clinic	Caries + Supernumerary Extraction	Oral + Intranasal (Moderate sedation); Biased coin design
**Canpolat et al. (2016) (62)**	Turkey	Randomized Clinical Study	n = 60	3 - 9y	25 M / 35 F	ASA I–II	University Dental Clinic	Extractions (IV sedation)	Compared IV Ketamine, Propofol, and Ketamine+Propofol
**Ghabchi et al. (2025) (63)**	Turkey	Retrospective 10-year Review	n = 96 (128 visits)	4 - 12y	63 M / 33 F	ASA I–II	University Pediatric Clinic	Extractions, Endo, Restorative, Surgery	162 procedures; Extractions (66%); 10-year record review
**Talukdar et al. (2025) (64)**	India	Retrospective Comparative Study	NR	Pediatric	NR	Anxious children	Dental Clinic	Routine Pediatric Procedures	Compared MID, DEX, and MID+KET protocols
**Patel et al. (2018) (65)**	India	Randomized Controlled Trial	n = 44	4 - 9y	NR	ASA I (Uncooperative)	Dental College Clinic	Pediatric treatments under sedation	Intranasal vs Oral Dexmedetomidine; 4 dosing groups
**Jeong et al. (2025) (32)**	Republic of Korea	Retrospective (5-year)	n = 1248	4.46y	805 M / 443 F	Mixed (ED Trauma)	Pediatric Emergency Dept	Closure, Splinting, Extraction, Pulp	Sedation rate 25.4%→29.2%; 348 sedated pts
**Peerbhay & Elsheikhomer (2016) (66)**	South Africa	Triple-blind RCT	n = 118	4 - 6y	NR	ASA I (Anxious)	Emergency Dental Clinic	Extractions	IN Midazolam (0.3 vs 0.5 mg/kg); Pulse oximetry
**Ansari et al. (2018) (67)**	Iran	Crossover Double-blind RCT	n = 23	2 - 6y	17 M / 6 F	ASA I (Frankl 1)	University Dental Clinic	Pulpotomy, Restoration, Extraction	Melatonin vs Midazolam; Two visits per child
**Chen et al. (2023) (68)**	Taiwan	Retrospective (BIS vs non-BIS)	n = 206	2 - 8y	NR	ASA I–II	Outpatient Dental Clinic	Restorations, Crowns, RCT, Extractions	IV Propofol (TCI); BIS monitored (n = 113)
**Mozafar et al. (2018) (69)**	Iran	Randomized Crossover RCT	n = 18 (36 sessions)	3 - 6y	9 M / 9 F	ASA I (Frankl 1 - 2)	Hospital Dental Clinic	Restorative (2 visits)	Video-rated behavior; Crossover design
**Moore et al. (2019) (70)**	USA	Retrospective Comparative	n = 1856	21mo–13y	~928 M / 928 F	ASA I–II (BMI < 35)	Tertiary Pediatric Clinic	Restorative, Extractions (1h)	IOGA vs Oral sedation; Success/Safety comparison
**Alhaidari et al. (2022) (71)**	Saudi Arabia	Randomized Crossover RCT	n = 32	3 - 6y	18 M / 14 F	ASA I (Uncooperative)	University Dental Hospital	Restorations, Pulp, Crowns, Extractions	Fentanyl quality improvement; Crossover design
**Chen & Tanbonliong (2018) (72)**	USA	Retrospective Cohort	n = 271	2 - 13y	148 M / 123 F	ASA I–II (Fearful)	University Outpatient Clinic	Comprehensive Dental Treatment	Comparison of 2 morphine-based oral regimens
**Yousif et al. (2025) (73)**	Iraq	Double-blind RCT	n = 40	5 - 10y	9 M / 31 F	ASA I (Anxious)	University Dental Clinic	Pulpotomy, Pulpectomy, SSC	Dexmedetomidine vs Ketofol IV; Endo focus
**Singh et al. (2025) (74)**	India	Crossover Clinical Study	n = 54	3 - 9y	31 M / 23 F	ASA I–II	Pediatric Dental Clinic	Bilateral Restorative Treatment	Temperament-based analysis
**Dubey et al. (2024) (75)**	India	Randomized Crossover RCT	n = 47	3 - 9y	NR	ASA I (Frankl 2)	Dental Hospital / Minor OT	Extractions, Pulpectomy, Restorative	1-week washout; Same patients for both regimens
**Nathan (2022) (31)**	USA	Retrospective Cohort (35y)	n = 2610 (visits)	3 - 7y	NR	ASA I–II (Anxious)	Private Pediatric Clinic	Restorative & Surgical care	CH–H doses with/without Meperidine comparison
**Van Anh et al. (2025) (76)**	Vietnam	Single-arm Intervention	n = 32	3 - 16y	21 M / 11 F	ASA I–II (Gag reflex)	Hospital Pediatric Dept.	RCT, Extractions, Restorations, SSC	100% completion; Stable vitals; No AE
**Hussien et al. (2022) (77)**	Egypt	Double-blind RCT	n = 40	5 - 10y	33 M / 7 F	ASA I (Uncooperative)	Hospital Dental Unit	Pulpotomy, Pulpectomy, SSC	IV sedation (Anesthesiologist supervision)
**Jaikaria et al. (2018) (78)**	India	Triple-blind RCT	n = 34	3 - 9y	NR	ASA I (Frankl 1 - 2)	Minor OT	Extractions, Pulpectomy, Restorative	Oral sedative combinations comparison
**Mehran et al. (2018) (79)**	Iran	Double-blind Crossover RCT	n = 30	2 - 6y	14 M / 16 F	ASA I (Frankl 1)	Hospital Pediatric Clinic	Pulp therapy on similar teeth	Self-control design; Two visits per child
**Rehman et al. (2021) (80)**	India	Randomized Controlled Trial	n = 30	2 - 5y	25 M / 5 F	ASA I (Uncooperative)	Hospital Pediatric Unit	Endodontics (Maxillary Incisors)	Oral Midazolam premedication in both groups

### 3.3. Study Design Architecture

The review demonstrates a sophisticated methodological landscape. Many studies, particularly those from Iran and India, used randomized controlled trials (RCTs) with crossover designs, strengthening internal validity by using patients as their own controls. While smaller RCTs focused on efficacy, large-scale studies, notably Nathan et al. (n = 2,610) (31) and Jeong et al. (n = 1,248) (32), provide robust data for detecting rare RAEs. This combination of rigorous RCTs and large retrospective cohorts strengthens the overall evidence base for assessing both efficacy and safety.

### 3.4. Sample Size and Statistical Power

Sample sizes varied substantially, ranging from 14 to over 2,600 participants. Studies of novel interventions, such as intranasal dexmedetomidine, typically included 30 to 60 subjects to maintain adequate statistical power. A notable methodological shift is observed from single-center, moderate-sized cohorts toward longitudinal analyses of large-scale hospital registries. This transition enhances the epidemiological understanding of adverse-event patterns and supports a more robust safety assessment across diverse pediatric populations.

### 3.5. Demographic Profiling

The 3 - 6-year age group is the most frequently studied cohort, representing the "critical window for dental anxiety" and higher physiological vulnerability due to narrower airways. The inclusion of outliers, infants under 2 (33) and adolescents over 10 (34), enhances the external validity of the findings across the pediatric spectrum. Most studies report a balanced (50/50) sex distribution, indicating that biological sex is not a determining factor in sedation modality selection or the incidence of RAEs.

### 3.6. Clinical Risk Assessment

While most participants were classified as ASA I and II, the review includes high-risk populations, such as children with asthma (35), Down syndrome (36), autism spectrum disorder (ASD), and neurological impairments (37, 38). Recent research shows a paradigm shift toward identifying high-risk subgroups, with an increased clinical focus on body mass index (BMI) and tonsillar hypertrophy. These factors are now recognized as critical predictors for understanding the mechanisms of upper airway obstruction during pharmacological sedation.

### 3.7. Clinical Settings and Infrastructure

Research was predominantly conducted in academic pediatric dental departments or hospital-based clinics (e.g., Albaker (39) and Kip et al. (40)), ensuring an "anaesthetic safety net" with specialists and resuscitative equipment. In contrast, private-practice studies (e.g., Rienhoff et al. (41)) favor short-acting agents such as oral midazolam because of their wider therapeutic indices. Recent trends (e.g., Liu et al. (38)) show increasing use of minor operating theaters (minor OT) for deeper sedation. This reflects a clear clinical demarcation between office-based and hospital-based protocols, dictated by drug dosage and patient systemic status.

### 3.8. Clinical Interventions and Procedural Complexity

Sedation is primarily reserved for an "invasive triad": pulpotomy, extractions, and stainless-steel crowns, treatments characterized by high-decibel noise, physical pressure, and long durations. While minor procedures (e.g., fissure sealants) are less common, their inclusion highlights that dental phobia can necessitate sedation regardless of clinical complexity. A significant emerging trend is full-mouth rehabilitation in a single session. This shift leads to extended sedation durations, which the literature identifies as an independent risk factor for respiratory fatigue and potential airway obstruction.

### 3.9. Qualitative Synthesis and Clinical Observations

Clinical notes reveal critical safety patterns. Vašáková et al. highlight the need for pharmacological reversal (flumazenil) for unpredictable paradoxical reactions, even under strict guidelines (42). Studies by Sado-Filho (33) and Wu et al. (37) show a 9 - 10.2% treatment-discontinuation rate, reflecting a "safety culture" that prioritizes aborting procedures over hazardous dose escalation. Notably, recent research identifies a 79-minute duration cut-off, beyond which respiratory risks increase exponentially (38, 43). Findings also emphasize that respiratory safety depends more on meticulous patient selection (BMI and tonsillar status) than on dosage alone.

### 3.10. Strategic Synthesis of Findings

A holistic evaluation of Table 1 reveals a multi-layered landscape. Layer I (methodological maturity): research has evolved from case reports to rigorous crossover RCTs and large-scale cohorts, providing high statistical power and a reliable foundation for clinical guidelines. Layer II (vulnerable populations): focus has shifted toward high-risk cohorts (obesity, airway obstruction, systemic comorbidities), aiming to optimize safety for the most challenging patients. Layer III (the setting-safety paradox): while hospitals provide advanced infrastructure, clinical challenges such as coughing or movement are driven by procedural invasiveness (surgical stimulus) rather than by environment or expertise. This suggests that procedural intensity remains a primary determinant of sedation stability.

### 3.11. Pharmacological Landscape: Dose Dynamics and Paradigm Shifts

Table 2 reveals a shift from the gold-standard midazolam (0.5 mg/kg PO; 0.2 - 0.3 mg/kg IN) toward dexmedetomidine (1 - 3 µg/kg), especially in 2020 - 2025 studies. This transition prioritizes "cooperative sedation" without the respiratory depression seen with benzodiazepines. Concurrently, a dose-sparing strategy is evident in synergistic combinations such as ketofol or triple cocktails (e.g., Albaker et al. (39)), reducing individual drug toxicity while enhancing overall safety.

**Table 2.** Drug Interventions and Respiratory Outcomes

Author (Year)	Drug (name)	Dose (mg/kg or total)	Route	Drug Combinations	Respiratory Monitoring Method	Observed respiratory adverse events (RAEs)	Severity	Management / Intervention	Final Outcome	Authors’ Recommendations
**Vašáková et al. (2020) (42)**	Midazolam syrup	0.5 mg/kg (Max 12 mg)	Oral	None (Mixed with syrup)	Pulse Oximetry, HR, BP	None (SpO_2_ > 95%)	None	Flumazenil (for paradoxical reactions)	Discharged (~2h)	0.5 mg/kg dose; Pulse-ox + Flumazenil backup
**Albaker et al. (2022) (39)**	Diazepam, Meperidine, Hydroxyzine	DIA: 0.28, MEP: 1.94, HYD: 1.53	Oral	Diazepam + Meperidine + Hydroxyzine	Pulse Ox, HR, RR, BP (AAPD)	None (1 tachycardia event)	Mild	O_2 for tachycardia	Safe Discharge	Essential monitoring; Polypharmacy is effective
**Hammadyeh et al. (2019) (44)**	Ketamine, Atropine, Dexmedetomidine	KET: 5, ATR: 0.01 / DEX: 3 µg/kg	Oral	G1: KET+ATR, G2: DEX	Pulse Ox, HR, BP (Every 10 min)	None (SpO_2_ > 97%)	None	None	Safe; DEX had faster recovery	Oral DEX: comparable efficacy, faster recovery
**Alzein et al. (2022) (45)**	Sevoflurane/ Propofol	SEVO: 8%→2 - 3% / PROP: 2 mg/kg→100 - 150µg	SEVO: Inh. / PROP: IV	Oxygen only	Pulse Ox, Capnography, ECG, NIBP	3 desaturations (SpO_2_ < 92%)	Mild	O_2 + Airway maneuvers	Safe Discharge	Mandatory monitoring for both agents
**Thakur et al. (2021) (46)**	Midazolam + Ketamine	A: 0.2+5 / B: 0.3+3 / C: 0.4+2	Oral (Honey)	Midazolam + Ketamine combinations	Pulse Ox, HR, BP (15 - 20 min intervals)	None (SpO_2_ ≥ 93%)	None	None (1 vomiting/hallucination)	Safe Discharge	Group B (0.3+3 mg/kg) most successful
**Hammadyeh et al. (2019) (47)**	Dexmedetomidine, Ketamine, Atropine	DEX: 1µg/kg + 0.2µg/h / KET: 2 mg/kg + ATR: 0.01	Intravenous	Ketamine + Atropine (Group K)	Continuous SpO_2_, HR, BP	None	None	None	Both safe; DEX: better behavior & faster recovery	Dexmedetomidine is more effective than KET+ATR
**Kip et al. (2019) (40)**	Sevoflurane, Ketamine, Propofol, Midazolam, Fentanyl	Varied (Ketamine IM/IV; Propofol bolus; Ketofol 1:1)	IV / Inhalation	Multiple (e.g., Ketofol; Sevoflurane + N_2O)	Continuous SpO_2_, BP, HR	Resp. depression 1.15%; Bronchospasm 0.7%	Mild to Moderate	Observation; No intubation	Safe recovery without sequelae	Ketofol reduces nausea/resp. depression; Continuous monitoring
**Ansari et al. (2018) (48)**	Midazolam, Atropine, Promethazine, Ketamine	MID: 0.5 + ATR: 0.25 / PROM: 1 (Premed) + KET: 2	Oral / IV	Crossover: MID+ATR vs. Promethazine	Continuous HR, BP, RR, SpO_2_	None (No hypoxia/apnea)	None	None	Safe recovery; Minor N/V	Promethazine effective as MID; Lower vomiting; Similar safety
**ElKhatib et al. (2024) (49)**	Dexmedetomidine, Midazolam	DEX: 5 or 3 + MID: 0.3 / MID: 0.5	Nebulized	DEX ± MID vs. MID alone	SaO_2, HR, BP continuously	Mild transient bradycardia (DEX); No desaturation	Mild	None	Stable respiration; Full recovery	DEX alone: better cooperation and easier completion than MID
**Rienhoff et al. (2022) (41)**	Midazolam	0.4 mg/kg (Max 7.5 mg)	Oral	None	Pulse Oximetry (SpO_2_, HR)	None reported	Mild/Transient	Continuous observation	Safe sedation; 6.1% discontinued 1st session	Midazolam + Hypnosis effective; ≤2 sessions recommended
**Wu et al. (2023) (37)**	Dexmedetomidine, Midazolam, Sevoflurane, Propofol	DEX: 2µg/kg IN; MID: 0.5 mg/kg PO; SEV (Induction); PROP: 2 - 3µg/ml	IN / PO / Inh. / IV	Dex/Midaz Premed; SEV (if needed)	SpO_2_, EtCO_2_, RR, BP, HR, ECG, BIS	35 cough; 18 desat < 95%; 25 hypoxemia ≤ 90%	Minor	Suction; Head-tilt; O_2 mask (7 cases)	All recovered < 30s; No serious events	Longer TX & cough ↑ risk; Desaturation most common
**Binh et al. (2025) (50)**	Midazolam	0.3 mg/kg or 0.6 mg/kg	Oral	None	Continuous HR & SpO_2_ (T0–T5)	None reported	None	Not required	Both doses safe; No hypoxia	0.6 mg/kg: better cooperation & longer sedation
**Sado-Filho et al. (2021) (33)**	Dexmedetomidine, Ketamine	DEX: 2.5 µg/kg / DK: DEX 2 + KET 1	Intranasal	Dex alone vs. Dex+Ketamine	Continuous HR & SpO_2_ (ASA)	One desaturation (88%); Vomiting episodes	Minor	Supplemental O_2 (for desaturation)	Similar efficacy; DK: longer recovery (1.3×)	DEX alone: fewer AEs and faster recovery
**Mehran et al. (2017) (51)**	Midazolam, Ketamine	Midazolam: 0.2 mg/kg; Ketamine: 0.5 mg/kg	Intranasal	None (Crossover design)	HR, BP, RR, SpO_2_ (T0–T4)	Desaturation (SpO_2_ < 90%) reported	Minor	Monitoring only	Both effective; KET: less crying & more sleep	Both agents effective for dental sedation
**Patel et al. (2025) (52)**	Midazolam, Dexmedetomidine	MID: 0.3 mg/kg; DEX: 1.5 μg/kg	Intranasal (Atomized)	None	Pulse Oximetry, HR, SBP, DBP	MID: 3 cases SpO_2_ = 94%; DEX: None	Mild, Transient	No treatment needed	DEX: 92% completion & lower HR/BP	IN DEX: safe/effective for anxious children
**Ghajari et al. (2016) (53)**	Midazolam	0.3 mg/kg or 0.5 mg/kg	Oral	Midazolam + Hydroxyzine (1 mg/kg)	SpO_2_, HR, RR (Alborz B9)	None (SpO_2_ remained normal)	None	Not needed	Both doses safe; Vitals normal	Both doses similarly effective and safe
**Abdulhamid et al. (2016) (35)**	Chloral hydrate	65 mg/kg (Single dose)	Oral	± Nitrous oxide (50%)	Continuous SpO_2_ & HR	SpO_2_ < 95% (3/24); Resp. depression (2/24)	Mild	O_2 supplement; TX interruption; ED observation	Safe recovery; No hospitalization	Consider alternatives due to respiratory risk
**Joshi et al. (2020) (54)**	Ketamine, Propofol, Dexmedetomidine	KP: KET 1 + PROP 1 / KD: DEX 1 + KET 1	IV	Midazolam premed; O_2 (3 L/min)	ECG, HR, BP, SpO_2_ (Every 5 min)	None (SpO_2_ > 95%)	None	Routine monitoring only	Adequate sedation; KD better quality	KD superior; Both stable and safe for IV
**Preethy & Somasundaram (2022) (55)**	Midazolam, Nitrous oxide	MID: 0.3 mg/kg / N_2O: 30 - 70%	IN (MAD) / Inhalation	None	Continuous SpO_2_, HR, RR, BP	N_2O: 5 vomiting; MID: Sneezing/Coughing	Mild	Routine monitoring only	Both methods safe; MID faster onset	IN Midazolam: safe alternative to N_2O
**Janiani et al. (2024) (56)**	Dexmedetomidine, Midazolam, Nitrous oxide	DEX: 1 μg/kg / MID: 0.3 mg/kg / N_2O: 30 - 50%	IN (Atomized) / Inhalation	None	Pulse Oximetry (SpO_2_), HR	MID: 64% coughing, 1 desat (94%); DEX: 14% coughing	Mild	Routine observation	All effective; MID deeper than DEX	MID: effective N_2O alternative; DEX: slower onset
**Hamod et al. (2022) (36)**	Dexmedetomidine, Midazolam	DEX: 1 μg/kg / MID: 0.2 mg/kg	Intranasal	None	SpO_2_, RR, PR, BP (Every 5 min - AAPD)	None (No significant difference in SpO_2_)	None	No intervention required	Stable vitals; Both effective in Down Syndrome	Both agents safe for children with Down syndrome
**Arnaout et al. (2025) (57)**	Midazolam	0.3 mg/kg	Intranasal / Buccal	None	Vital signs monitoring (General)	None reported	None	None	Both routes effective (Success > 80%)	Buccal & IN MID are effective for behavior management
**Zouaidi et al. (2022) (24)**	Midazolam, Meperidine, Hydroxyzine, Diazepam, Ketamine	Varied (IM Ketamine; Oral combos)	Oral / IM / Inh. (N_2O)	Multiple Oral combos (e.g., MEP+HYD; MID+HYD)	Continuous vitals; TROOPS classification	Low SpO_2_ (0.4%); Lung mucus (0.3%); Laryngospasm (0.1%)	Mild to Moderate	Suction; Positive pressure O_2; Extended monitoring	No deaths; Very low serious AE incidence	Standardized AE tracking (TROOPS) improves safety
**Yinger et al. (2024) (34)**	Triazolam	0.125 - 0.50 mg	Oral	Nitrous oxide (50%) for all	HR + SpO_2_ (Every 5 min)	SpO_2_ < 93% (n = 4); HR anomalies	Mild and Transient	No reversal; No airway intervention	Zero severe events/admissions	Triazolam is likely safe for mild–moderate sedation
**Razavi & Malekianzadeh (2022) (58)**	Midazolam, Propofol	MID: 0.5 mg/kg PO + PROP: 2 mg/kg IV + Infusion	Oral + IV	Oxygen via nasal cannula	ECG, NIBP, Pulse Oximetry	Laryngospasm (n = 5); Hypoxia 90 - 94% (n = 17); 1 repeated desat	Mild to Moderate	PPV; Bag-valve-mask; Airway maneuvers; Suction	99.6% success; No intubation; No PONV	Deep sedation safe with anesthesiologist & monitoring
**Unkel et al. (2021) (59)**	Dexmedetomidine, Midazolam, Hydroxyzine, Nitrous Oxide	DEX: 3µg/kg IN / MID: 0.5 - 0.7 mg/kg PO / HYX: 1 mg/kg PO	Intranasal + Oral	All groups with N_2O (50%)	BP, SpO_2_, HR, RR, EtCO_2_, ECG	1 Bradycardia (DEX); Hypotension events (All groups)	Minor	Self-corrected; No intervention required	Safe discharge; No apnea or obstruction	IN DEX + N_2O is safe and comparable to MID regimens
**Liu et al. (2025) (38)**	Dexmedetomidine, Propofol, Sevoflurane	DEX: 2µg/kg IN; PROP: 4 - 10 mg/kg/h (TCI)	IN → IV	Sevoflurane (for rescue induction)	SpO_2_, EtCO_2_, BIS (50 - 70), ECG, BP	Hypoxemia 8.6%; Choking cough 12.3%	Mild (↑ risk in Tonsillar hypertrophy)	Jaw thrust; Dose reduction; Suction; O_2 3 L/min	100% completion; No morbidity	Safe GA alternative; Airway screening essential
**Gomes et al. (2017) (60)**	Ketamine, Midazolam	IN: KET 4 + MID 0.2 / OR: KET 4 + MID 0.5 / OR: MID 1	Intranasal / Oral	3-arm Comparison	Continuous HR & SpO_2_ (Planned)	Not reported (Study Protocol)	N/A	N/A	N/A	Study Protocol; Aims to compare safety and efficacy
**Kocaoğlu et al. (2025) (43)**	Propofol, Midazolam, Lidocaine	MID: 0.05 mg/kg IV; PROP (TCI): 2 - 5µg/ml; LIDO: 0.5 mg/kg	IV	Propofol TCI + O_2 (2 L/min)	SpO_2_, EtCO_2_, BIS, HR, BP	Hypoxemia 10.3%; Bradycardia 1.8%	Mild to Severe (WHO)	Jaw-thrust; Chin-lift; Modify depth; Bag-mask	99.6% completion; Complications manageable	Hypoxemia risk ↑ with dose, time, & tonsillar hypertrophy
**Wang et al. (2023) (61)**	Esketamine, Midazolam, Sevoflurane	Midazolam: 0.5 mg/kg PO + Esketamine: 1.99 mg/kg IN	Oral + Intranasal	Sevoflurane (for rescue)	SpO_2_, HR, BP (Every 5 min)	0% Desaturation; Tachycardia 8.3%; N/V 8.3%	Mild, Transient	Observation; Rescue SEVO if needed	88.3% success; Mean wake-up: 89.4 min	Safe noninvasive outpatient sedation (2 - 6 years)
**Canpolat et al. (2016) (62)**	Ketamine, Propofol	K: 1 / P: 1 / KP: 0.5 + 0.5 (mg/kg)	Intravenous	Single drug vs. Combination	ECG, HR, MAP, SpO_2_, EtCO_2_, RR, Capnography	Resp. depression (3 cases in Propofol group); Tachycardia	Mild, short-term	Supplemental O_2 and observation	Propofol: fastest recovery; KP: highest satisfaction	Propofol alone caused more respiratory depression
**Ghabchi et al. (2025) (63)**	Nitrous oxide, Oxygen	30 - 50% N_2O; 100% O_2 (Pre/Post)	Inhalation (Nasal mask)	Monotherapy	HR, SpO_2_, BP (Continuous)	None reported (128 sessions)	None	No intervention required	Safe and effective for dental anxiety	100% procedural success rate
**Talukdar et al. (2025) (64)**	Midazolam, Dexmedetomidine, Ketamine	Not reported (Retrospective)	IV or Oral (Not specified)	Three protocols compared	Observation of Resp. depression & Agitation	MID: 12%; MID+KET: 18%; DEX: 4 - 5%	Mild (Highest in combo group)	Not stated (No major complications)	DEX is safest and most effective	Authors recommend future RCTs
**Patel et al. (2018) (65)**	Dexmedetomidine	2, 2.5, 4, 5 µg/kg	Intranasal / Oral	None	SpO_2_ BP, HR (Every 10 min)	None reported	None	No intervention required	IN DEX: faster onset & better depth than Oral	IN DEX is more effective than Oral with no AEs
**Jeong et al. (2025) (32)**	Ketamine, Midazolam	Ketamine: 1.24 - 2.08 / Midazolam: 0.1 (mg/kg)	IV (ED Sedation)	Ketamine alone vs. Ketamine + Midazolam	Continuous SpO_2_; Hypopnea/Apnea monitoring	Hypopnea (2.87%); Apnea (0.29%); Emesis; Hypersalivation	Mild to Moderate	O_2 (Mask/Prong); Flumazenil; BVM; Suction	Combo group: more respiratory AEs	Close monitoring recommended; Ketamine alone safer
**Peerbhay & Elsheikhomer (2016) (66)**	Midazolam	0.3 mg/kg vs. 0.5 mg/kg	Intranasal (MAD)	None	Pulse Oximetry	One case SpO_2_ drop to 90% (≈1%)	Mild	Observation only	Both doses safe; 0.5 mg/kg better behavior control	INM 0.3 - 0.5 mg/kg is safe; 0.5 mg/kg more effective
**Ansari et al. (2018) (67)**	Melatonin, Midazolam, Ketamine, Atropine	0.5 mg/kg (Oral) + IV protocol	Oral (Preme) / IV	Midazolam vs. Melatonin before IV KET+ATR+MID	HR, RR, BP, SpO_2_ (q15 min)	No significant respiratory events	Mild transient SpO_2_ drop (MID only)	Standard monitoring only	MID: deeper sedation/satisfaction; more N/V. Melatonin: fewer side effects	Midazolam preferred for efficacy; Melatonin safer
**Chen et al. (2023) (68)**	Propofol, Midazolam, Ketamine, Chloral hydrate, Fentanyl, Alfentanil	TCI PROP (Mean 341 ± 135 mg)	IV	Premed: MID/KET/CH; Rescue: Fentanyl/Alfentanil	Pulse Ox, NIBP, EtCO_2_, BIS	Hypoxia, Apnea, Cough (lower in BIS group); Bronchospasm	Mostly Mild to Moderate	Repositioning; Suction; Mask ventilation; 1 Intubation per group	Lower RAEs & faster discharge with BIS	BIS + TCI improves airway safety and recovery
**Mozafar et al. (2018) (69)**	Midazolam, Promethazine	MID: 0.5 mg/kg / PROM: 1 mg/kg	Oral	Combined with N_2O-O_2 (50%)	Pulse Oximetry (SpO_2_, HR q10 min)	Lowest SpO_2_ = 91% (Both groups)	Mild transient desaturation	O_2; Routine monitoring	Both safe; MID better behavior and less crying	Midazolam + N_2O preferred for quality
**Moore et al. (2019) (70)**	Chloral hydrate, Meperidine, Hydroxyzine, Midazolam, Diazepam, Sevoflurane, Propofol, Fentanyl	Standard clinical doses	Oral / Inhalation / IV	Multidrug Oral vs. TIVA or Inhalation GA	Standard monitoring; SpO_2_, EtCO_2_	Obstruction 18%; Emergent Succinylcholine 2.9%; Intubation conversion 8.4%	Mostly Mild-Moderate; 1 serious AE	O_2; Succinylcholine (for laryngospasm); Intubation	99.5% completion; 0.2% serious AE rate	IOGA (Anesthesia-led) is safe and improves completion
**Alhaidari et al. (2022) (71)**	Midazolam, Fentanyl	MID: 0.7 mg/kg (Oral); FEN: 1 µg/kg (IN)	Oral + Intranasal	Oral Midazolam + IN Fentanyl	Continuous SpO_2_, HR, BP	Transient desaturation (1.6%); No apnea	Mild	Head tilt; Suction; Reassurance	No reversal needed; Safe completion	Combination improves sedation & behavior
**Chen & Tanbonliong (2018) (72)**	Morphine sulfate, Midazolam, Diazepam, Hydroxyzine, Nitrous Oxide	Morphine: 0.5 - 0.7 mg/kg	Oral	Morphine + Mid/Dia + Hyd + N_2O/O_2	Pulse ox; Pretracheal stethoscope; BP	Transient desat (2.2%); Wheezing/Asthma (0.7%)	Mild to Moderate	Repositioning; Albuterol; Flumazenil (1 case)	Success > 80%; No serious sequelae	Morphine regimens effective with minimal AEs
**Yousif et al. (2025) (73)**	Ketamine, Propofol (Ketofol), Dexmedetomidine	Ketofol: KET 2 + PROP 4 (mg/ml); DEX: 2 μg/kg	IV	Ketofol vs. Dexmedetomidine alone	Pulse oximetry, RR, ECG, NIBP	RR fluctuations (Ketofol); Bradycardia (Dex)	Mild to Moderate	Dose adjustment; Close monitoring	Both effective; Dex: more stable respiration	Dex preferred for better respiratory stability
**Singh et al. (2025) (74)**	Nitrous Oxide, Oxygen	Up to 50% N_2O	Inhalation	Nitrous Oxide / Oxygen	Pulse oximetry, Pulse Rate (PR)	None (SpO_2_ ≥ 95%)	None	Routine monitoring only	Both titration methods safe	Rapid titration beneficial for negative temperament
**Dubey et al. (2024) (75)**	Ketamine, Midazolam, Dexmedetomidine	KET: 7 mg/kg / MID: 0.3 mg/kg + DEX: 3 µg/kg	Intranasal	IN Ketamine alone vs. IN MID-DEX	SpO_2_, Pulse Rate, BP (Continuous)	None reported	None	Routine monitoring only	KET: faster onset, recovery, and discharge	IN Ketamine preferred for efficacy, safety, and acceptability
**Nathan (2022) (31)**	Chloral hydrate, Hydroxyzine, Meperidine	CH: 25 - 50 mg/kg; MEP: 1 - 2 mg/kg	Oral	CH-H ± Meperidine	Pulse oximetry; Ventilation assessment	Rare desaturation; Somnolence	Mostly Mild	Verbal/physical stimulation; Rare Naloxone use	Higher success & fewer restraints with MEP	Lower CH doses with MEP: safer and more effective
**Van Anh et al. (2025) (76)**	Nitrous Oxide, Oxygen	30 - 40% N_2O (Max 50%)	Inhalation	None	SpO_2_, RR, BP, HR	None reported	None	O_2 titration; Routine monitoring	100% completion; High cooperation; Stable vitals	N_2O/O_2: safe and effective alternative to GA
**Hussien et al. (2022) (77)**	Ketamine, Propofol (Ketofol), Dexmedetomidine	Ketofol: KET 2 + PROP 4 (mg/ml); DEX: 2 µg/kg load	IV	Ketofol vs. Dexmedetomidine alone	SpO_2_, RR, HR, NIBP, ECG	No respiratory depression; ↑ RR variability in Ketofol	Mild	Dose titration; Rescue Propofol if needed	Both effective; Dex: more stable respiration	Dex preferred for respiratory stability
**Jaikaria et al. (2018) (78)**	Midazolam, Ketamine, Dexmedetomidine, Fentanyl	MID: 0.3; KET: 5 (mg/kg); DEX: 2; FEN: 3 (µg/kg)	Oral	MK vs. DF vs. DK combinations	Pulse oximetry	None reported	None	Continuous monitoring only	MK success: 72.8%; DF: 58.3%; DK comparable	Oral MK and DK are effective combinations
**Mehran et al. (2018) (79)**	Midazolam, Chloral hydrate, Promethazine	MID: 0.4 mg/kg + CH: 50 mg/kg or PROM: 5 mg/kg	Oral	Midazolam + CH vs. Midazolam + PROM	Pulse oximetry, BP, PR (q15 min)	No hypoxia (Lowest SpO_2_ = 94%)	Mild	Observation only	MID+CH: better sedation depth and cooperation	Prefer Midazolam + Chloral hydrate for better cooperation
**Rehman et al. (2021) (80)**	Propofol, Dexmedetomidine	DEX: 1 µg/kg; PROP: 1 mg/kg bolus + infusion	IV	Propofol ± Dexmedetomidine	Continuous SpO_2_, RR, HR, NIBP	Desaturation in Propofol-only group (3/15); None with DEX	Mild, transient	Supplemental oxygen; Monitoring	Successful sedation; Reduced Propofol dose with DEX	Dexmedetomidine is a safe and effective adjunct to Propofol

### 3.12. Routes of Administration: From Conventional to Innovative Delivery

The oral (PO) route remains common because of its simplicity, but recent shifts toward mucosal atomization devices (MAD) (e.g., Patel (52) and Janiani et al. (56)) highlight a preference for bypassing first-pass metabolism. The intranasal (IN) route appears superior for uncooperative patients, offering a less traumatic alternative to IV access. Innovations such as nebulized dexmedetomidine (49) further reflect the pursuit of "stress-free" induction and improved patient compliance.

### 3.13. Respiratory Monitoring Modalities: From Oxygenation to Ventilation

Continuous pulse oximetry (SpO_2_) remains the universal "gold standard," used in 100% of the included studies. However, the systematic adoption of capnography (EtCO_2_) and bispectral index (BIS) monitoring is observed in studies employing deep-sedation protocols or propofol (e.g., Liu et al. (38); Kocaoğlu et al. (43)). This highlights a critical clinical evolution: as sedation depth increases, the monitoring paradigm shifts from assessing oxygenation (via SpO_2_) to ensuring adequate ventilation (via EtCO_2_). Additionally, the use of a pretracheal stethoscope, as noted in several studies, underscores the continued relevance of acoustic monitoring as the most immediate clinical method for detecting early signs of airway obstruction.

### 3.14. Characterization of Observed RAEs

Table 2 reveals a distinct risk distribution across agents. The midazolam profile is generally uneventful or limited to transient desaturations (SpO_2_ > 90%), confirming its respiratory stability. In contrast, propofol and deep sedation show the highest rates of adverse events (apnea/hypopnea), with Kocaoğlu et al. (43) reporting a 10.3% hypoxemia rate, highlighting a narrow safety window. The ketamine profile features minimal respiratory depression but carries a risk of hypersalivation-induced laryngospasm at high doses. Finally, the intranasal route often triggers coughing and sneezing, reaching 64% in Janiani et al. (56); while not systemically dangerous, these reactions cause significant procedural interruptions.

### 3.15. Clinical Management and Rescue Interventions

Most RAEs were successfully managed with supplemental oxygen (2 - 3 L/min) and physical maneuvers (e.g., head-tilt/chin-lift, jaw-thrust). This indicates that pediatric dental complications are predominantly obstructive (mechanical collapse) rather than the result of central respiratory depression. However, the recorded need for bag-valve-mask (BVM) ventilation or agents such as succinylcholine (for laryngospasm, per Moore et al. (70)) underscores the need for crisis preparedness. Such interventions emphasize the critical requirement for advanced airway-management skills, particularly when intravenous-based sedation is used.

### 3.16. Authors' Recommendations

Analysis reveals three safety-oriented trajectories. First, continuous multimodal monitoring is a non-negotiable cornerstone of pediatric patient safety. Second, dexmedetomidine is identified as the superior agent for respiratory stability because of its minimal impact on ventilatory drive compared with benzodiazepines. Finally, 2024 - 2025 research emphasizes mandatory preoperative screening for tonsillar hypertrophy, shifting the focus toward risk mitigation through meticulous patient selection to prevent complications rather than managing them intraoperatively.

### 3.17. Integrated Synthesis of Findings (Pharmacological and Safety Dynamics)

Axis I (pharmacological risk stratification): a distinct risk hierarchy exists. Midazolam offers the lowest rate of severe events but carries a risk of sedation failure. Conversely, propofol ensures procedural completion through deep sedation but shows the highest rates of hypoxemia (up to 10.3%) and apnea. Meanwhile, dexmedetomidine has emerged as a "game changer," resolving the paradox between sedation depth and ventilatory safety through remarkable respiratory stability even at higher dosages.

Axis II (evolution of monitoring paradigms): high-impact studies (e.g., Liu et al. (38); Chen et al. (68)) indicate a shift from passive to active monitoring. While SpO_2_ remains the standard, capnography (EtCO_2_) and BIS are becoming the new benchmarks, allowing pre-hypoxia detection of respiratory depression, a clinical revolution in mitigating complications before they manifest systemically.

Axis III (dominance of physical maneuvers): a critical finding is that manual dexterity (e.g., jaw-thrust, suctioning) proved more frequent and effective than reversal agents (e.g., flumazenil). This underscores that events are predominantly obstructive (anatomical) rather than central (chemical), making airway maintenance clinically superior to manipulating drug chemistry.

## 4. Conclusions

### 4.1. Discussion

Table 1 reveals a transition from traditional monotherapies to multimodal protocols. Despite its frequent use, midazolam as a standalone agent often yields suboptimal behavioral outcomes. Preethy and Somasundaram (55) support this observation, reporting no significant differences in sedation across various midazolam delivery routes. Thus, modifying the administration route alone fails to improve behavior or safety, underscoring the need for synergistic drug combinations to address the limitations of benzodiazepine monotherapy.

Table 1 demonstrates an increasing shift toward dexmedetomidine (DEX) and combination regimens for uncooperative pediatric patients. This aligns with Haridoss et al. (81), whose review found that midazolam provides the shortest recovery (mean difference: 19.1 min), whereas DEX provides superior, high-quality sedation. These findings corroborate our Table 1 results, identifying DEX as highly successful. Clinicians must therefore navigate the trade-off between midazolam’s rapid recovery and DEX’s advantage of high-quality sedation without respiratory depression.

The synthesis indicates a shift toward combination therapy to reduce dosages and RAEs. Swaminathan et al. (82) confirm that combining midazolam with ketamine or dexmedetomidine improves onset and behavioral success and outperforms monotherapy. However, Marques et al. (9) highlight low-quality evidence for midazolam–nitrous oxide combinations. This evidence gap reinforces the need for the stringent monitoring protocols identified in Table 2 to maintain safety.

Managing children with ASD remains challenging because of higher risks of aggression during induction and secondary RAEs. Alyahyawi et al. (83) suggest combining nitrous oxide with oral benzodiazepines for ASD, consistent with the protocols in Table 1. However, Vallogini et al. (84) emphasize precise dose titration to prevent overdose and RAEs. This caution mirrors our findings, identifying accurate dosing as pivotal in preventing oxygen desaturation in this vulnerable population.

Table 2 shows substantial heterogeneity in RAE frequency and severity. Romagnolo et al. (85) note that complications depend on specific agents and patient traits, mirroring our findings of RAEs ranging from coughing to transient apnea. Consequently, non-pharmacological interventions are essential; Gao and Wu (2) argue that behavior management reduces pharmacological load and adverse events. This suggests that many documented RAEs may be preventable through optimized behavior management as a first-line defense against sedative risks.

A pivotal finding is the high success of midazolam–ketamine in enhancing efficacy while mitigating complications. Gharavi et al. (86) support this, reporting that this regimen outperforms monotherapies for pediatric sedation and pain relief. This confirms our Table 2 data, suggesting that combination protocols enable dose-sparing effects that minimize respiratory risks. Furthermore, the 84% success rate reported by Rathi et al. (87) is consistent with the high success rates for multimodal protocols extracted from our tables.

Table 2 indicates that, although oxygen desaturation is the most concerning, complications such as bradycardia, hypotension, and postoperative nausea and vomiting (PONV) also occur. Abuokal et al. (88) found no significant differences in these non-respiratory events across groups, consistent with our data. Additionally, Taneja and Jain (89) reported no significant disparities in induction outcomes or sedation depth. This underscores that procedural success often depends more on clinician expertise in physical and behavioral management than on the specific pharmacological agent used.

Evidence quality remains suboptimal in several domains. Oza et al. (90) assigned “extremely low certainty” to the available evidence because of limited trials and unconventional parameters, mirroring our observation of discrepant RAE definitions. Additionally, Khole et al. (91) found no significant differences (P = 0.64) and a high risk of bias in most studies. These limitations necessitate cautious interpretation of our results and underscore the need for high-standard randomized clinical trials with standardized outcome reporting.

The synthesis from Tables 1 and 2 identifies four key themes. The anatomy-pharmacology nexus: respiratory safety depends on the interaction between patient characteristics (Table 1) and pharmacological profiles (Table 2). Even agents considered safe, such as dexmedetomidine, can cause problems in patients with anatomical risks (e.g., tonsillar hypertrophy), making preoperative screening as important as dose titration. Shift to precision management: pediatric dentistry is moving from single-agent midazolam toward “monitored polypharmacy with capnography.” This shift prioritizes respiratory quality alongside behavioral success, ensuring physiological stability during treatment. Novel administration routes: intranasal atomization acts as a “missing link,” reducing physiological stress by avoiding IV cannulation; by minimizing crying and agitation, this route may indirectly prevent RAEs such as laryngospasm. Safety versus efficacy paradigm: high-safety protocols (e.g., nitrous oxide) are suitable for short procedures in cooperative patients, whereas for complex, full-mouth rehabilitation in uncooperative children, intravenous combinations (e.g., ketofol) are becoming the standard because of their high success rates, provided that advanced hospital monitoring is used.

### 4.2. Study Limitations

Several limitations were identified. First, evidence certainty in trials is often low or very low. Second, substantial heterogeneity in RAE definitions hindered direct comparisons and limited generalizability. Finally, the lack of long-term and oral-health-related quality-of-life (OHRQoL) data necessitates caution in generalizing the findings to broader clinical contexts.

### 4.3. Concluding Remarks

Pediatric sedation has transitioned from traditional single-agent approaches to sophisticated multimodal protocols and safer, next-generation drugs. Combination regimens enhance clinical success and minimize severe respiratory risks through dose-sparing effects. Modern sedation is now a comprehensive risk-management system in which drug selection, administration route, and patient anatomy interact to safeguard respiratory integrity. Ultimately, success requires integrating behavior management with pharmacological protocols to ensure physiological safety and improve the child’s long-term quality of life and psychological well-being. These findings support individualized sedative selection and monitoring based on patient risk profiles.

## Data Availability

The dataset presented in the study is available on request from the corresponding author during submission or after publication. The data are not publicly available due to privacy concerns and consent restrictions.
